# Use of flow cytometry method to detect contaminations of platelet suspensions

**DOI:** 10.1007/s11274-024-04030-x

**Published:** 2024-05-30

**Authors:** Mehtap Bolat, Hüseyin Hatipoğlu, Mehmet Köroğlu, Hande Toptan, Mustafa Altındiş

**Affiliations:** 1 Sakarya University Health Sciences Institute, Sakarya, Turkey; 2 Medical Microbiology Laboratory, Sakarya Training and Research Hospital, Sakarya, Turkey; 3 Faculty of Medicine, Department of Medical Microbiology, Sakarya University, Sakarya, Turkey

**Keywords:** Transfusion medicine, Blood components, Bacterial contamination of platelet suspension, Flow cytometry, Shelf life

## Abstract

In this study, it was aimed to investigate bacterial contamination in apheresis platelet suspensions (APS) by automated blood culture system and flow cytometry method (FCM).

33 spiked APS each using 11 bacterial strains (5 standard strains, 6 clinical isolates), were prepared in three different dilutions (1–10, 10–50, 50-100 cfu/mL), incubated in two different temperatures (35–37 °C and 22–24 °C) and different incubation times (18–96 h) evaluated by FCM. This three different dilutions were also inoculated into special platelet culture bottles (BacT/ALERT® BPA) and loaded into the blood culture system. Additionally 80 APSs routinely prepared in the Transfusion Center were evaluated by both FCM and the blood culture system. Platelets were lysed by freeze-thaw method.

All spiked samples were positive with BacT/ALERT® BPA in 12–18 h. In 96 h incubation at 22–24 °C, the presence of bacteria was detected by FCM in all other samples (31/33) except low dilutions (1–10 and 10–100 CFU/ml) of *K.pneumoniae* standard strain. In the 35–37 °C, the presence of bacteria was detected by FCM in all samples (33/33) after 48 h of incubation. In routine APS one sample detected as positive (*Bacillus simplex*) with BacT/ALERT® BPA and no positivity was detected by FCM.

The freeze-thaw method, which we have optimized for the lysis of platelets, is very practical and can be easily applied. The BacT/ALERT® system has been found to be very sensitive in detecting bacterial contamination in PSs. Flow cytometry method has been found to be successful, fast, easy to use and low cost in detecting bacterial contamination in PSs.

## Introduction

Treatment with blood and blood products is not only life saving but also important in terms of infectious diseases in blood recipients (Reesink et al. 2008). The World Health Organization defines safe blood as blood that does not pose any danger or disease to the person it is given, and does not contain infectious agents or harmful foreign substances (Kocazeybek 2003). However, despite all the precautions taken today, bacterial contamination continues to be an important risk, especially for patients receiving platelet suspension transfusion (Levy et al. 2018).

Blood components can be obtained from whole blood or by the “apheresis” method (Hitzler 2014). The superiority of the apheresis method is that it provides a higher amount of desired blood product compared to whole blood collection (Hitzler 2014). With the transfusion of blood components, different microorganisms can be transmitted apart from the pathogens routinely screened for (Heroes et al. 2020). Cases of sepsis caused by bacteria transmitted from blood and blood products are rare. However, since the mortality risk is high in this type of sepsis cases, it is of great importance for human health (Juffermans et al. 2011). The incidence of transfusion-related bacterial infections and sepsis cases has decreased thanks to the use of aseptic techniques in the donation stage (whole blood collection and apheresis), cold storage of blood and blood components, and the use of sterile closed systems during the collection/separation of whole blood (Blajchman and Goldman 2001).

Platelet suspensions (PS) are the most risky blood product in terms of bacterial contamination when compared to other blood components. Since it is stored at room temperature and contains sufficient nutrient media for bacteria, it becomes easier for bacteria to grow (Blajchman and Goldman 2001, Korte 2011). Screening of PS for bacterial contamination will significantly reduce transfusion-induced bacterial infections in the recipient (Murdoch and Greenlees 2004). It has been reported in the literature that bacterial contamination was detected in one of approximately 2000–3000 PSs (Dreier et al. 2009).

Storage times of PS vary between countries. Studies on increasing the storage period of PS from 5 days to 7 days by ensuring the safety of use are becoming widespread (Caram-Deelder et al. 2016). One of them is the investigation of bacterial contamination in these products. PS, which have been shown to be free of bacterial contamination, have a shelf life of 7 days (Munksgaard et al. 2004).

BacT/ALERT® (Biomerieux, France) and Becton Dickinson® (Becton Dickinson, USA) automated blood culture system and special platelet culture bottles have been approved by the FDA (Food and Drug Administration) in the USA. Screening of bacterial contamination in PS using these bottles and systems has been used in the last 20 years and is becoming increasingly common. Some publications emphasize that screening for bacterial contamination reduces the frequency of transfusion-related bacterial infections (Munksgaard et al. 2004).

There are also studies in the literature that bacterial contamination can be demonstrated by flow cytometry method, thus reducing the infection rate and extending the storage period (Vollmer T., et al.2011). However, as far as we could scan, these studies are very few. In this study, it was aimed to investigate the bacterial contamination in PS by using special platelet culture bottles compatible with the automated blood culture system and flow cytometry method, thus increasing the shelf life of PS from 5 days to 7 days.

## Materials and methods

### Ethics committee approval, samples and bacterial strains

Approval for this study was obtained from the Sakarya University Faculty of Medicine Ethics Committee (71,522,473/050.01.04/297).

33 Spiked apheresis platelet suspensions (APS) (samples prepared by adding bacterial suspensions at various concentrations to APS) and 80 non-spiked APS samples were included in the study.

In the simulation study, standard bacterial strains (*Escherichia coli* ATCC 25,922™, *Klebsiella pneumoniae* ATCC 700,603™, *Pseudomonas aeruginosa* ATCC 27,853™, *Enterococcus faecalis* ATCC 29,212™, *Staphylococcus aureus* ATCC 29,213™) and clinical isolates of the same bacterial species found in our laboratory’s culture collection were used. In addition to these, *Staphylococcus epidermidis* clinical isolate was also used in simulation studies. The clinical isolates found in the culture collection of our laboratory were identified by mass spectrometry (VITEK MS®, bioMérieux, France) method.

### Preparation of spiked samples

All bacterial strains were inoculated on 5% sheep blood agar plates and incubated at 35 ± 2 °C for 16–18 h. Bacterial suspensions (BS) were prepared at 0.5 McFarland density for each bacteria using the photometric method (DensiCHEK plus, bioMérieux, France). Bacterial suspensions were prepared by serial dilutions. Two PS + BS were prepared by taking 1 ml of bacterial suspensions and adding 1 ml of PS. Thus, PS + BS was obtained with a final concentration of 1–10, 10–100, 100–1000 CFU/ml.

These prepared platelet + bacteria suspensions (PS + BS) were taken into sterile tubes and incubated in a platelet agitation device for 16–18 h at room temperature and in an incubator (35–37 ºC) and the samples were analyzed by flow cytometry. The incubation period was extended to 96 h for samples that were FCM negative at these incubation times. As a result, FCM analyzes were performed at 18, 48 and 96 h. Simultaneously, two ml of the prepared TS + BS samples were inoculated into BacT/ALERT® BPA culture bottles (bioMérieux, France) and loaded into an automatic blood culture device (BacT/ALERT®, bioMérieux, France). The positive signal times of the PS Culture bottles were recorded.

### Analysis of non-spiked apheresis platelet samples

In the other part of the study, APS samples taken routinely in the Transfusion Center of our hospital were inoculated in a BacT/ALERT® BPA culture bottle (bioMérieux, France) for bacterial contamination and examined in an automatic blood culture device (BacT/ALERT®, bioMérieux, France) and flow cytometric method.

5–10 ml of sample was taken from the sampling part of the PS bag taken by apheresis, inoculated into the culture bottle and placed in the automated blood culture system. Samples that were incubated in the blood culture device for five days and did not receive a positive signal were considered negative in accordance with the manufacturer’s recommendations. 1 ml of each routine PS sample was incubated separately in the incubator and at room temperature. Samples were analyzed by flow cytometry on days 1, 3 and 5. In addition, when a positive signal was obtained from the PS culture bottle, FCM analysis was performed from the samples that were separated for FCM and left to incubate (room temperature and in an incubator).

### Sample preparation and method optimization for FCM analysis

Before starting the analysis of the samples examined within the scope of the study, preliminary trials were made and the method to be applied in FCM analyzes was optimized. At this stage, it was observed that the separation of bacteria and platelets was not possible with FCM without the lysis of platelets or the use of additional fluorescent dyes. It was observed that 2 different lysis solutions used in hematological analyzes in flow cytometric methods were insufficient in the lysis of platelets. Two different lysis solutions used in hematological and immunological flow cytometric analyzes were tried to lyse platelets. However, for bacterial flow cytometric analyzes in our study, these two solutions were found to be insufficient to lyse platelets. It was aimed to apply a platelet lysis method that can be easily used in every laboratory (Fukuda et al. 2020; Rodrigues et al. 2022; Kandoi et al. 2018). In our literature review, it was seen that there were a few studies using the freeze-thaw method to achieve platelet lysis. In these studies, platelets were subjected to lysis by the freeze thaw method for purposes other than flow cytometric analyses. In the experiments, it was observed that the lysis of the platelets was achieved by freezing and thawing the platelet suspensions 3 times. For the lysis of platelets, it was kept 3 times at – 80 ºC for 15–20 min and then waited until it dissolved at room temperature. With this method, the lysis of platelets was achieved and the centrifuge stage was started. In addition to the platelet suspension samples to be analyzed in this study, bacterial suspensions were also subjected to the freeze thaw method as positive control. In positive controls, it was observed that the freeze thaw method had a minimally negligible effect on the number of bacteria. This study aimed to demonstrate the presence of bacteria qualitatively.

Centrifugation was performed at various speeds and times in order to separate the lysed platelets from bacteria. As a result, the optimum centrifugation speed and time were determined as at 500 rpm for 3 min. After obtaining two parts as pellet and supernatant in the centrifuged sample, the supernatant part was discarded and the pellet was diluted 10 times with phosphate buffer and the staining process was started. In the experiments, the pellet was suspended with different dilution coefficients and as a result, it was seen that 10-fold dilution gave optimum results. After the pellet was suspended, experiments were carried out with various concentrations of fluorescent dyes and incubations at different times. Optimum results were obtained by staining 100 µl pellet suspension with 20 µl (150 µM/ml) Syto 9 dye (Thermo Fisher, USA) and 20 µl (400 µg/ml) propidium iodide (PI) (Thermo Fisher, USA) and incubating in the dark for 15 min. Afterwards, the mixture was loaded into the flow cytometry device and the gating processes and analyzes were performed. While analyzing the spiked samples, positive control (suspension containing only bacteria) and negative control (suspension containing only platelets) samples, which were subjected to the same procedures, were used and gating and analysis procedures were performed considering these results. Positive control (suspension containing bacteria only) samples were diluted to a final concentration of 10^6^ CFU/ml of each bacteria. While optimizing the platelet suspension FCM analysis method, the distinction between platelets and bacteria was prioritized. As a result of each experiment, it was observed that the analyzes were suitable and the above procedure was determined as the optimum method.

### FCM analyzes

Before flow cytometric analyses, device maintenance and quality controls were performed in accordance with the instructions in the BD Accuri C6 Flow Cytometer Instrument Manual and the BD Accuri C6 Software User Guide. Six-peak validation beads were used to verify the performance of flow cytometry. Fitting of the FSC-SSC distribution and other fluorescence (FL1, FL2, FL3 and FL4) peaks into the correct channels was ensured according to the manufacturer’s instructions.

**Fig. 1:**
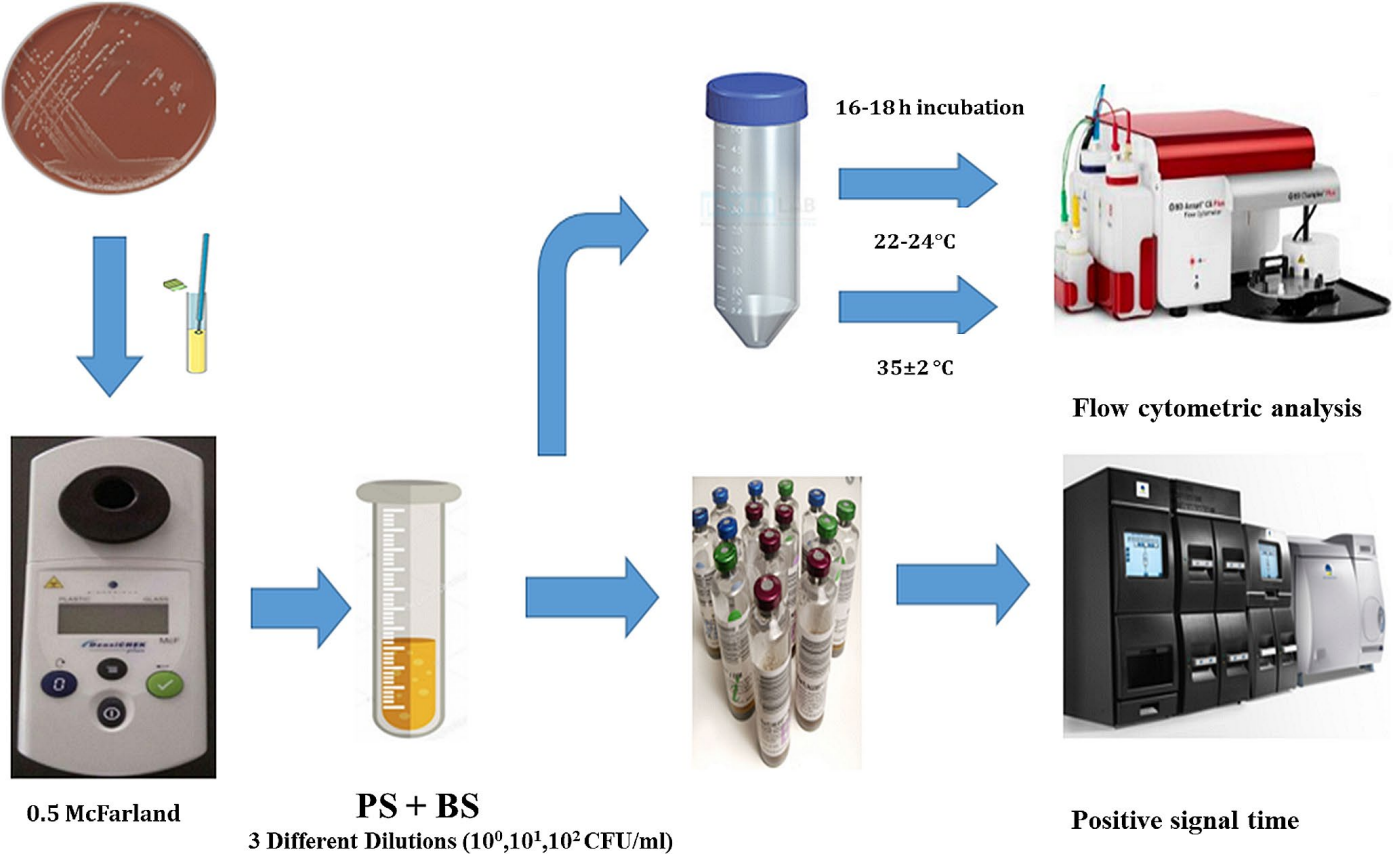
Analysis process of spiked samples. *PS*: platelet suspension, *BS*: bacterial suspension

**Fig. 2:**
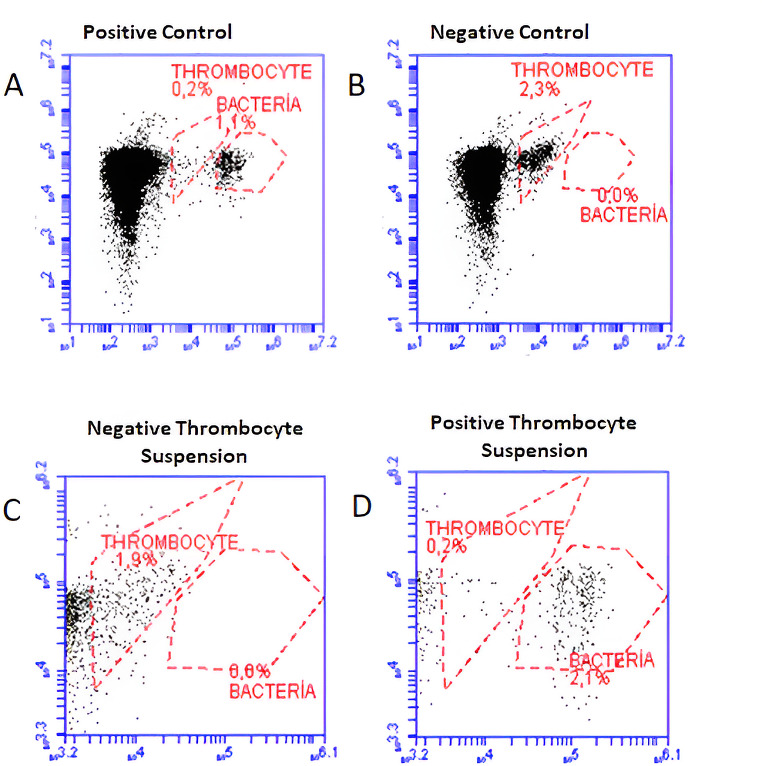
Dot blot image in FCM analysis. **A**: positive control **B**: negative control. **C**: uncontaminated thrombocyte (platelet) suspension. **D**: thrombocyte (platelet) suspension with bacterial contamination

For bacteriological analyzes, we used the FCM analysis protocol that we used in our previous study (Hatipoglu et al. 2022). As described in detail above, 1 ml was taken from all samples (spiked and non-spiked APSs), platelets were lysed, centrifuged, pellet suspension was prepared, fluorescent staining was performed and analyzed in a flow cytometry device (Accuri C6, Becton Dickinson, USA). A dot blot image of the positive control and negative control wells was recorded for each sample. The dot blot images of each spiked and non-spiked APS samples were compared with the dot blot images of the positive and negative control. The results obtained in FCM analyzes were evaluated by comparing with the automated platelet culture method (BacT/ALERT® BPA), which is accepted as the reference method in this study. The steps followed in the FCM study are presented in Fig. 1. Thrombocyte/platelet suspension’s gating procedures and graphic images performed on the FCM device are presented in Fig. 2.

## Results

Growth was detected in all spiked samples (*n* = 33, including standard strains and clinical isolates) and all dilutions during 12–18 h incubation period with BacT/ALERT® BPA platelet suspension-specific blood culture bottles (Table 1). In this study, the BacT/ALERT® BPA method was found to be 100% sensitive.

**Table 1:**
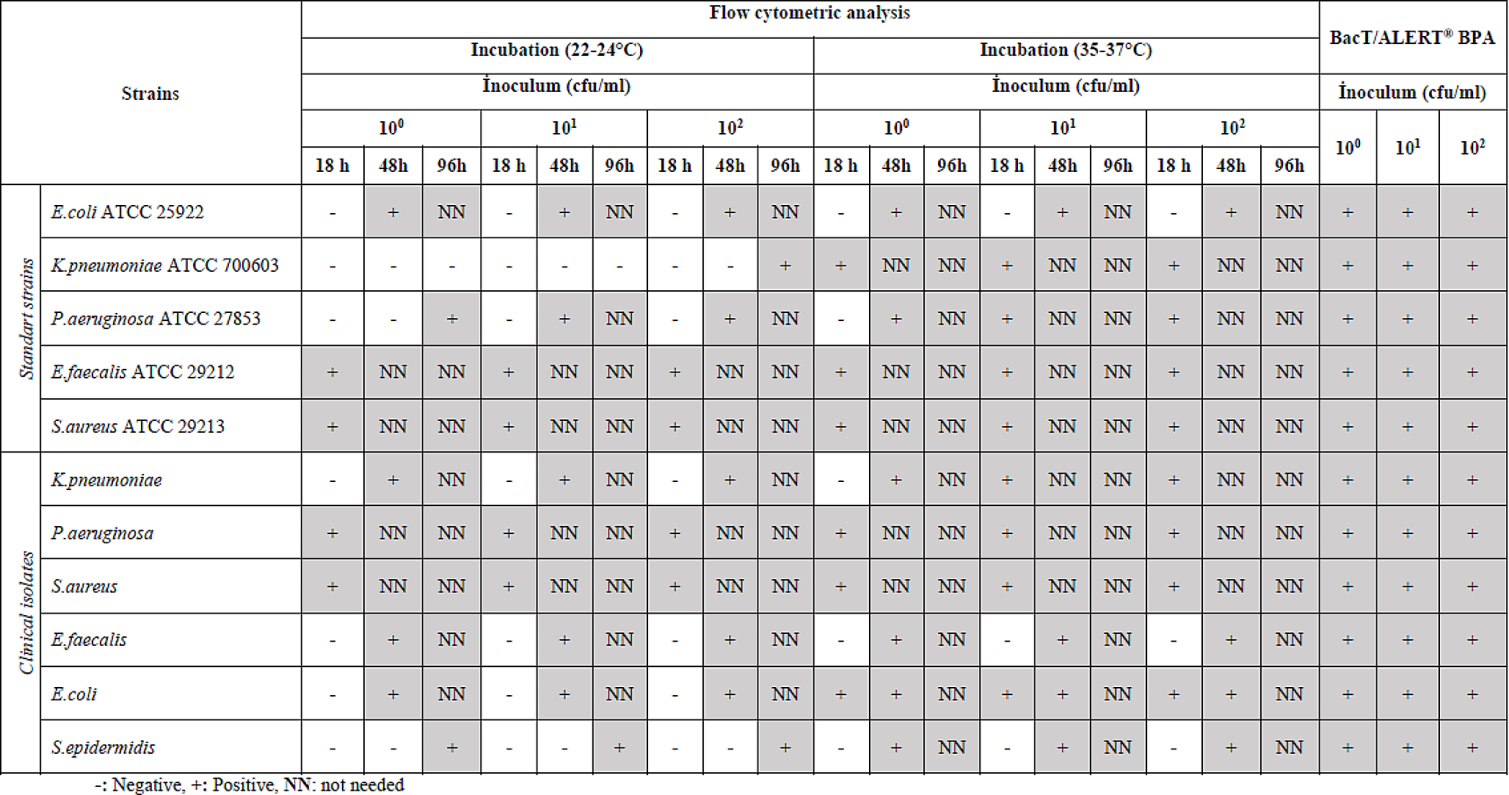
BacT/ALERT® BPA bottle and flow cytometric analysis results of spiked samples

In flow cytometric analyzes of spike samples prepared with standard strains after 18 h of incubation, the presence of bacteria could not be detected by FCM in all dilutions for *E. coli* standard strain at room temperature and in the incubator, and at room temperature for *K. pneumoniae* and *P. aeruginosa* standard strains. After 18 h of incubation of the *P. aeruginosa* standard strain in the incubator, the presence of bacteria could not be detected by FCM at a concentration of only 1–10 CFU/ml. When the incubation period was increased to 96 h, only *K. pneumoniae* bacteria could not be detected by FCM at room temperature and concentrations of 1–10 and 10–100 CFU/ml. The presence of bacteria was detected by FCM in platelet suspensions in all standard strains and all dilutions except the last mentioned (Table 1).

In flow cytometric analyzes of spike samples prepared with clinical isolates, bacteria could not be detected in platelet suspensions at room temperature and in the incubator in all dilutions for *S. epidermidis* and *E. faecalis* after 18 h of incubation. When the incubation period was increased to 48 h, *E. faecalis* could be detected at room temperature, while *S. epidermidis* remained undetectable. For the clinical isolate of *E.coli* and *K. pneumoniae*, FCM analyzes were negative in all dilutions after 18 h of incubation at room temperature. However, only 1–10 CFU/ml concentration of bacteria could not be detected for *K. pneumoniae* after 18 h of incubation in the incubator. When the incubation period was increased to 96 h, the presence of bacteria in platelet suspensions was detected in all clinical isolates and all dilutions (Table 1).

A positive signal was obtained in the BacT/ALERT® automated blood culture system in only one (1,25%) of the 80 non-spiked APS samples included in our study. The bacteria grown in this sample were identified as Bacillus simplex by the VITEK MS® system. In the flow cytometric analysis, no positivity was detected for all samples, including this sample, at room temperature and in the incubator (35 ± 2 °C) on the 1st, 3rd and 5th days.

## Discussion

Platelet suspensions pose a risk for transfusion infections due to room temperature storage conditions (Levy et al. 2018). For this reason, it is stated in national and international guidelines that platelet suspensions should be screened for the presence of bacteria in order to prevent transfusion-related bacterial infections and sepsis (Örüç et al., 2016). There are various methods used to determine whether there is bacterial contamination in platelet suspensions.

With the ability of devices to count bacteria in smaller structures, the increase in the number of lasers in devices, and the emergence of newly developed dyes, this method has been used more frequently in microbiology (Steen et al. 1982). In this study, the method of platelet culture bottles compatible with the automated blood culture device was taken as reference and it was aimed to detect bacterial contamination of platelet suspensions by flow cytometry before administration to the patient in a short period (in 1–2 h). Since the presence of skin flora members is frequently detected in the contamination of platelet suspensions, these bacteria were especially selected in studies where contamination was investigated (Agzie et al. 2019). The bacteria in our study were selected from the most common nosocomial infections agents and S. epidermidis, which is a skin flora member (Bereket et al. 2012).

When performing bacterial contamination analyzes in platelet suspensions with FCM, the background noise formed by platelets should be eliminated. It has been seen in the literature that platelets were subjected to lysis before FCM analysis (Dreier et al. 2009). Generally, various chemicals, centrifugal filtration, enzymatic method and ready-made commercial kits were used for the lysis of platelets (Dreier et al. 2009; Vollmer et al. 2011). These are often the factors that increase the cost. In studies where FCM analysis is performed without lysing the platelets, the background noise caused by the platelets is quite evident. (Lee et al. 2012). In our study, it was found that the FCM images obtained without platelet lysis were similar to those in this study. In addition, in FCM dot blot graphics, it was seen that background noise of platelets and images of some bacteria overlapped in the same area and bacteria could not be detected.

Lee et al. reported that the use of platelet suspension in small volumes increased the sensitivity in terms of bacterial contamination detection (Lee et al. 2012). In other words, if fewer platelets are present in the analysis, bacteria in suspension can be detected more easily in FCM analyses. We obtained similar findings in our preliminary studies. However, in this study, we aimed to develop a method that can be applied routinely, practical, low cost, standardized and can be done by all users without special training. Therefore, we eliminated background noise of platelets, as in previous studies, in order to make bacteria easily and more clearly visible in FCM analyses. As explained in detail in the Method section, the lysis of the platelets was ensured by freezing and thawing the platelet suspensions 3 times, and the method was optimized.

FDA-approved BacT/ALERT® culture bottles specific to platelet suspensions have been used in many studies for the detection and prevention of bacterial contamination (Jacobs et al. 2005; Levy et al. 2018). There are studies reporting that the transfusion of contaminated platelet suspensions is prevented by using this method, and according to these studies, the storage period can be extended from 5 days to 7 days in some countries. (Munksgaard et al. 2004; Korte 2011). However, Castro et al. argued that the BacT/ALERT® method alone would not be sufficient to increase the storage time of platelet suspensions from 5 days to 7 days and emphasized that an additional method is needed (Castro et al. 2005). In our study, the presence of bacteria was determined by the BacT/ALERT® method after 12–18 h of incubation in all of the spiked samples in all the dilutions we prepared. It can be said that this method is a very effective and sensitive phenotypic method. It has also been shown that it can detect the presence of even a small number of bacteria in less than 24 h. In this study, the performance of the FCM method was investigated with reference to this method.

Dreier et al. studied bacterial contamination with FCM (BACTIFLOW, Biomerieux, France) on spiked samples prepared with 11 standard bacterial strains. With standard strains, spiked samples were prepared at concentrations of 10^2^-10^3^ and 10^3^-10^4^ CFU/ml and analyzed with the Bactiflow device after incubation at 37 °C for 15 min and staining 30 °C for 12 min, and they were able to detect all of these bacteria with FCM. In this study, some commercial kits were used in the Bactiflow device. They reported that flow cytometry is a sensitive and inexpensive method that gives results in a short time (Dreier et al.2009). In this study, the standard strains used in common with our study were used in much higher concentrations. In addition, FCM analyzes were performed shortly after the bacterial suspension was prepared. No commercial kit was used in our study. However, the presence of bacteria at much lower concentrations could be detected by FCM.

In similar publications in the literature, when PS + BS is kept at 35 ± 2 °C, most of the bacterial species can be detected within the first 24 h, as is usually the case in our study (Mohr et al. 2006). However, in cases where the number of bacteria in the suspension is less than 10^2^, this time may not be sufficient. In our study, all of the bacterial species (standard strains; *E. coli, P. aeruginosa*, clinical strains; *K. pneumoniae, E. coli, S. epidermidis*) that could not be detected by FCM at the end of incubation under 18-hour incubation conditions could be detected in all dilutions after 48 h of incubation.

In this study, we found that 18 h of incubation at room temperature was insufficient for FCM analyzes to detect bacterial contamination in PS. Since room temperature is not the optimum temperature for bacterial growth, we may not be able to detect bacterial contamination with FCM because the division time of bacteria is prolonged. The number of bacteria considered clinically significant in many publications in terms of bacterial contamination has been reported as 10^5^ CFU/ml (Störmer and Vollmer 2014). As a matter of fact, in a few studies in the literature, it was emphasized that the incubation period at room temperature should be extended for FCM analyzes, similar to our data (Mohr et al. 2006). No significant difference was observed in the detection of gram-positive and gram-negative bacteria by FCM at room temperature and incubator 35 ± 2 °C.

In the literature review, it was seen that the most commonly used dye to detect bacterial contamination of platelet suspensions was thiazole orange. Thiazole orange is a fluorescent dye that binds to nucleic acids and stains both viable and non-viable cells. Therefore, all cells containing nucleic acid are stained easily, so it is difficult for some bacteria to separate from the platelet cluster when platelets are not lysed. In some studies, Fluorescein Diacetate (carboxyfluorescein diacetate and carboxyfluorescein diacetate succinimidyl ester), known as a vitality dye, was used (Dreier et al. 2012). This fluorescent dye does not fluoresce until it is degraded by functional cytoplasmic enzymes. It has been shown in studies that it makes it easier to detect bacteria in the dot bot image in FCM analysis, as it does not stain the lysed platelets (Dreier et al. 2009). In our study, Syto 9 and Propidium Iodide, which provide staining by binding to nucleic acids, were used. While Syto 9 stains both dead and living cells, Propidium Iodide can only stain dead cells. However, since nucleic acids from dead cells stain with both PI and Syto 9, they fluoresce more than live cells stained with Syto 9 alone. This facilitates the differentiation of nucleic acids from dead cells and live bacteria that cause bacterial contamination in FCM analysis.

Only one (1,25%) of 80 APS samples included in our study from routine studies had a positive signal in the BacT/ALERT® automated blood culture system. The bacteria grown in this sample were identified as Bacillus simplex by the VITEK MS® system. In the flow cytometric examination, no positivity was detected in all samples, including this sample, at room temperature and in the incubator on the 1st, 3rd and 5th days. It can be said that this bacterium did not grow in sufficient numbers in the 16–18 h incubation period by FCM and longer incubation times should be applied. In our study, it was determined that the incubation times of spiked samples should be kept longer at room temperature.

There is no standardization as to at what stage FCM analyzes should be used in routine practice to detect bacterial contamination in PS. APSs can be used any time for 5 days after preparation. Since FCM analyzes are concluded in a short time, it can be analyzed before PS is given to the patient to determine whether there is bacterial contamination. However, it is impractical to perform the FCM analysis in this way, since detection of contamination requires prolonged incubation at room temperature. In Mohr’s study, incubation in the incubator was found to be more advantageous in terms of detecting bacterial contamination (Mohr et al. 2006) The largest study in the literature in which bacterial contamination in PS with FCM was investigated and routinely used was the study by Müller et al. (Müller et al. 2015). In the study, they worked with FCM once from PS at the end of the 3rd day. They used the FCM method as the screening test and the blood culture bottle method as the confirmation method. These researchers used a commercial product with the same brand of flow cytometry device and lysis kits and set a limit value of 300 counts/ml for bacterial contamination with FCM. In their country they extended the shelf life of the PS by 2 days if it was found to be negative by FCM at the end of the 3rd day. Although Müller et al. have performed bacterial contamination analysis in more than 30 thousand PSs with FCM for about 2 years and reported that they work routinely, FCM is not widely used worldwide yet. It is not used routinely, partly because of its high LOD, partly due to its high cost and partly due to the lack of standardization. In the aforementioned publication and other existing publications in the literature, direct samples were taken from PS with a satellite bag and the study was carried out. No pre-incubation or pretreatment steps were used, except for the lysis of platelets. For the routine use of FCM in the detection of bacterial contamination in PS, it would be appropriate to take a sample with a satellite bag after the PS is taken and leave it to incubate at 37 °C for 5 days like a blood culture bottle. In line with the data we obtained in our other study (Hatipoglu et al. 2022) and as suggested by some researchers, in cases where there is no satellite bag, we think that pre-incubation of the sample taken from the PS for 1–2 h in an incubator at 37 °C would be useful in detecting the presence of bacteria with FCM. We recommend that researchers consider this process in their study design. In this way, at the end of the 5th day, the life of the negative PSs can be extended by 2 days by analyzing with FCM. However, we also think that the FCM method is a screening test. For this reason, we believe that it would be more appropriate to use the FCM together with the BacT/ALERT® automated blood culture system for the detection of bacterial contamination in PSs, rather than using it alone.

Since this is a preliminary study and method optimization, studies with more bacterial strains could not be performed. In addition, although we tried various incubation times, incubation of PS at 37 degrees for a few hours was not done, as in some studies. Our studies on this subject continue and we plan to carry out more comprehensive studies.

More studies with longer incubations and more bacterial strains are needed to make a general conclusion. However, in the light of this study, it can be said that bacterial contamination should be investigated at least in platelet suspensions, how important the issue is and should not be neglected.

The fact that more bacterial strains and samples could not be examined, incubation times could not be kept longer, and analyzes were made only in APSs can be counted as the limiting factors of this study.

In conclusion; The freeze-thaw method, was used for the first time in the literature in order to lysis of platelets in flow cytometric analysis. We have optimized this sample preparation method for the lysis of platelets and it is very practical and can be easily applied in the whole laboratory. The BacT/ALERT® system has been found to be very sensitive in detecting bacterial contamination in platelet suspensions. Flow cytometry method has been found to be successful, fast, easy to use and low cost in detecting and screening bacterial contamination in platelet suspensions. However, since the FCM method has not yet been fully standardized for this purpose, it should be used together with the blood culture bottle method even if used as a screening test.

## Data Availability

No datasets were generated or analysed during the current study.
